# Case of Injury of the Head, Followed by Loss of Taste and Smell

**Published:** 1824-08

**Authors:** J. Arnison

**Affiliations:** Surgeon, Alston, Cumberland.


ART. V.—*Case of Injury of the Head, followed by Loss of Taste and Smell.* By J. ARNISON, Esq. Surgeon, Alston, Cumberland.

AT a late hour on the night of Wednesday, the 9th of July, 1823, I was called to John Currah, of Ganigill, a man thirty-eight years of age, who had, a few hours previously to my seeing him, received a blow upon his head, by the fall of a piece of stone from the top of the shaft of a copper-mine, wherein he was working, a height of thirty-four yards. The stroke rendered him insensible; but, in a short time, he so far recovered from his insensibility, as to be drawn up to the shaft in the usual manner. I found him delirious, with a high degree of nervous excitement; pulse 72, and full; eyes contracted freely. By introducing my finger within the wound of the scalp, which was between three and four inches in length, I ascertained a fracture and depression near the lower posterior edge of the right parietal bone; he was also sick, and vomiting. I bled him to the amount of eighteen ounces, and put a temporary dressing upon the wound.

On the morning following, the delirium was much increased, pulse 66. At two P.M. I saw him again, in company with two professional gentlemen; the delay being occasioned by their residing at a distance. After a consultation, we agreed to apply the trephine; but our intention was wholly frustrated, the patient's delirium rendering it impossible to keep the head steady for performing the operation with safety.

On the 11th, we considered him a little more favourable, the delirium not being so violent, but still so as to prevent the operation. He continued in this state till the 15th, when he became more settled, rested quietly, and continued to improve slowly; in consequence of which, the use of the trephine was deemed unnecessary. He had been bled frequently, also had taken mercurial and saline cathartics, with diaphoretics. The wound appeared healthy, but was not wholly healed, till a small portion of bone exfoliated, which I removed from the wound on the 24th of October.

Six weeks after the occurrence of the accident, I was greatly surprised to hear he had lost all sense both of taste and smell. The first discovery he made of this was one evening whilst at supper, which he found devoid of taste: to satisfy himself, he took a table-spoonful of mustard, but this was also tasteless. That I might be fully convinced of the truth of his assertions, I requested him to call upon me the first time he came to Alston. I then gave him a glassful of brandy and a glassful of water, a little sugar and a little powdered ginger, of each of which he took a part, but did not feel the slightest taste they had: all were to him alike, except when his mouth was full of brandy, and his tongue came in contact with the upper part of his mouth, he felt that part a little warmer than when he had water in it; but, if repeated more than once, the brandy and water were both alike. I also gave him liquor of ammonia and assafœtida to smell at: the latter he returned, saying, "I cannot perceive any smell it has;" the ammonia, he said, was the strongest thing he ever felt; "it has made my eyes water, but I do not find any smell it has." He was, whilst labouring under delirium, very much averse to take any of the saline purgatives, which he would retain in his mouth, and, when he thought himself unobserved, would spit it out, even when given to him as water; and from this it may reasonably be concluded, that his sense of taste was at that time quite perfect.

To the present day, all things to him have the same taste, or rather no taste at all; neither does he enjoy that pleasure in satisfying the cravings of his appetite which he used to do: with these exceptions, his health is perfectly good, and he continues his former employment at the copper-mine, belonging to the commissioners and governors of Greenwich Hospital.

*Alston, Cumberland; June* 30, 1824.

